# FEA analysis of Normofunctional forces on periodontal elements in different angulations

**DOI:** 10.6026/97320630018245

**Published:** 2022-03-31

**Authors:** Bhavya Shetty, Ibrahim Fazal, Safiya Fatima Khan

**Affiliations:** 1 Department of Periodontics, Ramaiah University of Applied Sciences Faculty of dental Sciences, Bangalore, Karnataka, India

**Keywords:** Finite element analysis, Normofunctional load, Stress, Force inclinations

## Abstract

In the literature, the periodontal tissue reaction to dissimilar occlusal stress has been described, including clinical and histologic changes caused by stresses in periodontal structures. With respect to occlusal forces, periodontal assembly demonstrates varying adaptive capacity from individual to individual and period to period within the same individual. Unfortunately, these occlusal stresses are yet to be quantified. As a result, determining the effect of normal occlusal force on periodontal elements in various angulations is of interest. Based on CBCT images, one FEA of the maxillary First molar was created, consisting of tooth pulp, periodontal ligament (PDL), and alveolar bone; the effect of normal occlusal force on the pdl in alternate angulations was assessed. Occlusion will occur at three contact areas representing the centric occlusion contact points, each of which will share a 150 N force. The analysis was performed for four force inclinations (0, 22.5°, 45°, and 90°). Maximum stresses are observed in cases of 90-degree loading. These stresses, however, are insignificant and will not cause the periodontal components to rupture. These tensile stresses, which are concentrated in the apical and cervical regions, may obstruct blood flow, resulting in tooth decay or, in some cases, periodontal breakdown in PDL. There have been attempts to express numerical data of stress to be provided for normal and hyper function loads to simulate occlusal situations at various angulations that are known to be accountable for healthy and diseased periodontium.

## Background:

The role of occlusion in periodontal health and the effects of occlusal trauma on the periodontium leading to periodontal breakdown have been studied since years, but the results of research studies are still inconsistent and questionable. Numerous studies have evaluated the stress produced by occlusal forces within the tooth and supporting structures, however none of them have focused the same with a static load/stress with varying angulations. Finite element analysis is a numerical form of computer analysis using mechanical engineering that allows the stress to be identified and quantified within the structures constructed using elements and nodes [1]. Vandana et al.[2] were the first to report of distribution of stresses in PDL tissues (PDL, root, and alveolar bone) using a 3D FEM. Another study by Stones et al. [3] utilized a three-dimensional (3D) FEM model of natural central incisor to calculate maximum and minimum principal stresses in the PDL of maxillary central incisor with different bone heights. The adaptive capacity of periodontal structure (in relation to occlusal forces) differs significantly from person to person and even in the same individual at different time periods. The periodontal tissue reaction to variations in occlusal forces has been described in the literature wherein clinical and his to logic changes are discussed that is produced due to stresses in the periodontal structures. Unfortunately, these stresses are not quantified. Therefore, it is of interest to assess the effect of normal occlusal force on periodontal ligament in different angulations.

## Materials and methods:

The finite element analysis was performed on a personal computer (Pentium III, Intel) using ANSYS software, marketed by ANSYS Inc., USA and hyper mesh 13.0 is used for creating Fe-models. In this study, a 3D FEM of the anatomic size and shape of an average Indian maxillary first molar was constructed. Variable PDL widths were developed at different occlusogingival levels derived from the data of Coolidge. The use of these varying thicknesses makes the model more precise and realistic. The data of cone beam computed tomography (CBCT) of a healthy periodontium with no persistent periodontal pockets, clinical attachment level loss, absence of recession, no evidence of tooth wear, attrition, carious lesion or restorations in molars, periodontal diseases, malocclusion upon examination, in the outpatient of Ramaiah University of Applied Sciences- Faculty of Dental Sciences was selected.

## Inclusion criteria:

[1] Healthy individual.

[2] Healthy periodontal tissue.

[3] Intact dentition, no oral inflammation.

[4] Use CBCT to scan, collect 3D information of maxillary teeth, and obtain data in DICOM format.

## Exclusion criteria:

[1] Subject with systemic conditions.

[2] Subject with persistent periodontal pocket.

[3] Subject with clinical attachment level loss.

[4] Subject exhibiting gingival recession.

[5] History of periodontal intervention within the last 6 months.

## Method of collecting data:

A maxillary first molar was used to design a sample for FEA, consisting of tooth pulp, periodontal ligament (PDL), and alveolar bone, effect of normal occlusal force on pdl in alternate angulations was assessed on the images acquired by CBCT. Occlusion was at three contact areas representing the centric occlusion contact points, which equally shared a force of 150 N. Analysis was carried out for four force inclinations (0, 22.5°, 45°, 90°) Based on establishment of a finite element analysis model, using CBCT images, the effect of normal occlusal force on pdl in alternate angulations was assessed. In accordance with the evidence provided by Paolo M et al.,2002 simulating, conventional occlusal force of 150 N were applied as a distributed load to the occlusal surface of molar [4], Ravi Tejaswari Reddy et al. 2018 reported 150N as the normofunctional force [1]. Taking this evidence into account, the normal occlusal force was taken as 150N. Occlusion was assumed at three contact areas (Figure 1 - see PDF) representing the centric occlusion contact points, which equally shared a force of 100 N. The analysis was carried out for four force inclinations [5] (0, 22.5°, 45°, and 90°) (Dalia M et al. 2014) the stresses and tooth displacement were analyzed using color coding deformation and graphical animations. The tables comprised of numerical values of above information. Since, this study did not include any treatment with any patients, written informed consent and ethical clearance is not applicable for this study.

## Results:

3D tetrahedral elements were used for Fe-models, total nodes = 30838 and total Elements = 167089. Units Followed were displacement in 'mm', Force 'N', Stress 'MPa'. For applied loads and boundary conditions, the top portion (apical) of the alveolar bone is fixed in all directions, and an occlusal force is applied at three contact areas representing the centric occlusion contact points, which shared a force of 100 N equally (Figure 2a, 2b - see PDF). The study was conducted for four force inclinations (0, 22.5°, 45°, and 90°). Table 1(see PDF) lists the properties of the materials used for each periodontal element. Figure 3(see PDF) depicts the tooth displacement contours, which show that maximum movement occurs with a 90-degree load event and minimum movement occurs with a 0-degree load event. Figure 4(see PDF) depicts the displacement contour with colour coding, where it can be seen that the palatal movement of the teeth is greatest in the 90-degree angulation load case. The tooth movement increases as the angle-displacement ratio increases, i.e., when the angle-displacement ratio increases from 0degree, the palatal movement increases as well. (Table 2 - see PDF) Graphic depiction in Figure 5(see PDF) indicates that maximum stress in enamel is observed at the loading points on the cusps, maximum tensile stress of 35.23 MPa is observed with 90degree load event. Whereas, Figure 6(see PDF) depicts maximum stress in Dentine (Mpa), where it is observed at the red patches as shown, maximum tensile stress of 9.1MPa is observed with 90-degree load case, followed by 45 degrees then by 22.5 degrees and no or minimal stress provided by 0 degrees. Figure 7(see PDF) portray maximum stress in PDL is observed at the cervical region (red patches appreciated at buccal side), maximum tensile stress of 4.71MPa is observed with 90degree load case, followed by 45-degree load then 22.5 and shutting the stress in the end by 0 degree. Figure 8(see PDF) depicts the maximum stress in cortical bone and it is observed that this stress is mainly observed at the cervical region (red patches), maximum tensile stress of 5.42MPa is observed with 90deg load case and no/minimum stress at 0-degree case. It also shows the maximum stress in cancellous bone, and it is observed that at the root apex and cervical region (red patches), maximum tensile stress of 2.11MPa is observed with 90deg load case followed by 45-degree load.

When Von-Missus stress comparison was done between the different periodontal parameters at different angulations on a steady normofunctional load it was observed that in the enamel, maximum stress bearing was detected at 45-degree case which is 35.95 Mpa followed by 90-degree load. As for dentin, 22.5-degree angulation showed maximum load followed by 0-degree angulation. Surprisingly, for periodontal ligament it was observed that 45-degree angulation and 90-degree angulation showed same amount of maximum stress under 100N of load. For cortical as well as cancellous bone, 90-degree angulation showed maximum stress. (Table 3 - see PDF) For maximum principal stress variation; when 100N of load was induced at various angulations it was observed that maximum stress was detected in all the periodontal elements at 90-degree angulations, followed by 45-degree>>22.5 and finally the lowest for 0-degree angulation case. (Table 4 - see PDF)For maximum tooth displacement, on application of 100N force in 0-degree angulation 0, minimum tooth displacement was observed whereas maximum tooth displacement was observed at 90-degree angulation. However, this mobility is not enduring; the displacement recurs when the force applied is nullified. (Table 2 - see PDF).

## Discussion:

A harmonious relationship between the teeth, jaws, masticatory muscles, and temporomandibular joint (components of the stomatognathic system) contributes to periodontal health. Periodontal disease may develop if the interrelationship is disrupted. Paolo et al. [6] demonstrated that the load transfer is optimal for the model with neutral molar positions. Molar displacement, whether mesial or distal, results in less efficient load-transfer mechanisms, resulting in higher stresses to compensate for the horizontal shift of the bite force [7].PDL responses to physiologic loading are critical for understanding the tooth-support mechanism, loss of support due to periodontal pathology, and tooth and attachment apparatus reactions to orthodontic loading. As a result, determining the magnitude, nature, and direction of masticatory loads dissipated by the PDL is critical to understanding the attachment apparatus's biologic behaviour under normal and diseased conditions. Reddy et al. [1] used a 3D FEM to investigate the Von misses stresses in a natural model of the maxillary central incisor tooth, PDL, and alveolar bone at an angle of 50° to the long axis of the tooth on the palatal surface in a palatolabial direction, at the level of the middle third of the crown, and at different bone levels. Maximum stresses were found in the cervical region and, to a lesser extent, at the root apex. This result was consistent with our findings, which showed that maximum stresses were detected at the root apex and cervical region in periodontal ligament cortical and cancellous bone, and that maximum tensile stress was observed with a 90deg load case, followed by a 45deg load case with a stable force of 100N. Von Mises stresses were used in their study, which are a theoretical measure of stress used to estimate yield failure criteria in ductile materials and are also commonly used in fatigue strength calculations. Von Mises stresses are not ideal for studying compression and tension on a tooth because it is a brittle material. Therefore, the present study used minimum principal stresses to measure the stresses as it best represents the compression state of the stress.

Geramy and Faghini [7] studied the compression stresses in the labial site of the PDL in 3D FEM model of maxillary central incisor with normal to reducing alveolar bone heights. The highest stress levels were traced in the sub-cervical area, except for model of 8 mm of alveolar bone loss. An increase of compressive stress up to 17.13 times on the cervical and 9.9 times in the apical area was shown as compared with normal bone height model. Based on FEM analysis, 2.5 mm of alveolar bone loss can be considered as limit beyond which stress alterations were accelerated and the alveolar bone loss increases stress produced in PDL which is consistent with our study where it can be detected that there can be a spontaneous bone loss once there is sturdy force applied at 90deg angulation. Shalma Muneer et al.[8] concluded that at normofunctional load, the stresses were maximum on the mesial side near the cervical region for tooth (-10.93 MPa), for PDL (-4.06 MPa), for bone (-4.3 MPa) with normal bone levels; as the bone levels decreased the stresses increased and the stresses tend to concentrate at the apical region, which coincides with our study where it can be observed that at normofunctional load, maximum stress is observed at the cervical region and sometimes at the root apex when force of 100N is applied in 90 degree angulation (4.71 MPa) for PDL, (5.42 MPa) for cortical bone and (2.11 MPa) for cancellous bone with normal bone levels. Reducing the stresses in the PDL may provide a better condition for the tissues to continue its regenerative and physiologic functions. Therefore, when there is occlusal trauma, occlusal therapy may result in better healing and regeneration than without occlusal therapy. If the maximum allowable/permissible stress in the PDL is found, there can be a more accurate discussion in the findings of this study. Data provided by this study are in agreement with several studies, though not conducted by FEM, and also in disagreement with others, such as study by Comar et al. [9] where he concluded that inflammation was observed in both load bearing and non-load bearing teeth and this pressure creates a tension which could induce osteoclastic activity which might lead to resorption of the bone and might narrow the periodontal ligament space even if load would be restricted within is physiological limits, he also reported that rebound effects could be seen within the matter of 14 days. Evidence provided by Svanberg et al. [10] reported in beagle dogs, that if a consistent normal load within limits would be applied for a prolonged time it may lead to the progression of experimental periodontitis. There are no studies of tooth displacement occurring due to the normal occlusal load. However, from the available literature, the results of this study could be correlated to the finite element study by Tanne et al. [11] in which an orthodontic load of 100 gm was used to study the effects of root length and alveolar bone loss on patterns of initial tooth displacement which was found to be 1 mm for average root length of 13 mm. The advantage of FEM in this study is that we are able to show the changes in numerical stress values at normo-, hyper-, and hypo-occlusal loads. In the oral cavity, there are discrepancies in occlusal load on every tooth which depends upon the type of food consumed, physiologic activity performed, muscular action, and added parafunctional habits. The identification of stress values at different sites in a functional mouth is important to understand the role of occlusion on periodontal tissues. Further, the histologic evaluation of periodontal tissue changes should be correlated with stress values using FEA.

This study may have limitations, such as the assumption that a tooth is thought to be immobile in relation to the supporting bone, which is thought to be rigid, and the nodes connecting the tooth to the bone are thought to be fixed. This assumption will introduce some error; it should be noted that because bone remodeling (resorption and apposition) occurs as a result of compressive stress, the widened PDL would likely retain less compressive stress per unit area, limiting mechanical stimuli for further resorption; thus, this model can only represent the condition that initiates occlusal traumatism and not the dynamic changes that accompany lesion formation. The progress in the finite element analysis will be limited until better-defined physical properties for enamel, dentin, PDL, and cancellous and cortical bone are available. Despite its drawbacks, FEM can be considered a useful tool for visualizing stress in periodontal structures because the actual physical properties of the materials involved can be simulated. As a result, with the available computer knowledge, this method comes the closest to simulating the oral environment in vitro [11]. Furthermore, there are no quantitative guidelines to assist clinicians in making proper adjustments to ensure that stress in the supporting structures is evenly distributed. In this regard, the FEM has been tried, but with certain estimates and assumptions. As a result, more research is needed to link the effects of increased dynamic occlusal loads to changes in periodontal tissues.

## Conclusion:

Maximum stresses in the parts are observed with 90deg loading cases for the applied loads and boundary conditions. These stresses, however, are minor and will not result in fracture of the crown or periodontal elements. However, in PDL, tensile stress concentrated at the root and cervical regions may cause congestion of the arterial supply to the teeth, leading to tooth decay or, in some cases, periodontal breakdown. Future in vivo studies to assess the histologic effects of various occlusal forces are expected at this stage.
